# Improvement of malaria diagnostic system based on acridine orange staining

**DOI:** 10.1186/s12936-018-2214-8

**Published:** 2018-02-07

**Authors:** Masatsugu Kimura, Isao Teramoto, Chim W. Chan, Zulkarnain Md Idris, James Kongere, Wataru Kagaya, Fumihiko Kawamoto, Ryoko Asada, Rie Isozumi, Akira Kaneko

**Affiliations:** 1 Radioisotope Centre, Graduate School of Medicine, Osaka City University, 1-4-3, Asahi-machi, Abeno-ku, Osaka, 545-8585 Japan; 2 Department of Parasitology and Research Center for Infectious Disease Sciences, Graduate School of Medicine, Osaka City University, 1-4-3, Asahi-machi, Abeno-ku, Osaka, 545-8585 Japan; 3 Island Malaria Group, Department of Microbiology, Tumor and Cell Biology (MTC), Karolinska Institutet, Nobels väg 16, 171 77 Stockholm, Sweden; 4 Department of Parasitology and Medical Entomology, Faculty of Medicine, Universiti Kebangsaan Malaysia Medical Centre, 56000 Kuala Lumpur, Malaysia; 5 Nagasaki University Nairobi Research Station, NUITM-KEMRI Project, Nairobi, 00202 Kenya; 6 Institute of Tropical Disease, Kampus C Airlangga University, Surabaya, 60115 Indonesia; 7 Department of Radiology, Osaka Butsuryo College, 3-33, Ohtori Kita-machi, Nishi-ku, Sakai, Osaka Prefecture 593-8328 Japan; 8 Institute of Tropical Medicine, Nagasaki University, Nagasaki, 852-8102 Japan

**Keywords:** Staining, Acridine orange, Fluorochrome, Malaria diagnosis, LED, Interference filter

## Abstract

**Background:**

Rapid diagnosis of malaria using acridine orange (AO) staining and a light microscope with a halogen lamp and interference filter was deployed in some malaria-endemic countries. However, it has not been widely adopted because: (1) the lamp was weak as an excitation light and the set-up did not work well under unstable power supply; and, (2) the staining of samples was frequently inconsistent.

**Methods:**

The halogen lamp was replaced by a low-cost, blue light-emitting diode (LED) lamp. Using a reformulated AO solution, the staining protocol was revised to make use of a concentration gradient instead of uniform staining. To evaluate this new AO diagnostic system, a pilot field study was conducted in the Lake Victoria basin in Kenya.

**Results:**

Without staining failure, malaria infection status of about 100 samples was determined on-site per one microscopist per day, using the improved AO diagnostic system. The improved AO diagnosis had both higher overall sensitivity (46.1 vs 38.9%: p = 0.08) and specificity (99.0 vs 96.3%) than the Giemsa method (N = 1018), using PCR diagnosis as the standard.

**Conclusions:**

Consistent AO staining of thin blood films and rapid evaluation of malaria parasitaemia with the revised protocol produced superior results relative to the Giemsa method. This AO diagnostic system can be set up easily at low cost using an ordinary light microscope. It may supplement rapid diagnostic tests currently used in clinical settings in malaria-endemic countries, and may be considered as an inexpensive tool for case surveillance in malaria-eliminating countries.

**Electronic supplementary material:**

The online version of this article (10.1186/s12936-018-2214-8) contains supplementary material, which is available to authorized users.

## Background

Malaria remains an acute public health problem with annual estimates of 214 million new cases and 438,000 deaths [1]. There is an increasing demand for rapid diagnosis of malaria to prevent drug over-use and a resulting development of drug resistance. Rapid diagnosis is also desired in elimination settings to identify malaria hotspots and outbreaks, and detect infections among migrants. Currently available rapid diagnostic tests (RDTs) are lateral flow immuno-chromatographic devices that detect the presence of malaria parasite antigens, such as histidine-rich protein 2 (HRP2). However, they give only a qualitative result. Moreover, a high rate of false positives was reported in high transmission areas due to the persistent antigenicity of HRP2 [2].

For a quantitative result, Giemsa staining of thick blood films remains the gold standard but is time consuming. Rapid staining by acridine orange (AO), a fluorochrome dye, was extensively studied as an alternative approach to Giemsa staining [3]. Many variations of AO-staining protocols have been tried over the past half-century. Even in recent publications, there have been various rapid staining protocols with different AO concentrations: 1000 ppm (1 mg/ml) [4], 60 ppm [5], 20 ppm [6], and 100 ppm [7].

However, two remaining problems have prevented the application of AO staining for malaria diagnosis in endemic countries. One is that an epifluorescence microscope system is too expensive for most malaria-endemic countries. The other problem is AO staining inconsistency. The first problem was partially solved by a modified fluorescence microscope system using a light microscope and a halogen lamp [7, 8], but that system was inconvenient because of difficult focusing due to insufficient light intensity and unstable commercial power supply system [3]. The second problem was, although not often discussed, that “blood films were easily over- or under-stained” by AO, as noted by Craig and Sharp [5]. This staining inconsistency would often occur even in a film, as was stated for supravital staining of blood that “preselection of good quality regions is frequently necessary” [9]. AO films must therefore be prepared very carefully [5]. In a usual field setting, only one or two films are obtained from each participant, whose age and haematocrit may vary from person to person, resulting in staining problems because of difficulty to maintain “the proper thickness of films” [5].

To overcome these difficulties of AO diagnostic system, two modifications were applied: (1) a low-cost and brighter light-emitting diode (LED) light together with mobile batteries; and, (2) novel AO staining protocol, which produces an effective gradient of stain concentration. This improved AO diagnostic system was tested in malariometric surveys on islands in Lake Victoria, Kenya in August 2015. It showed stable and high performance in on-site field study.

## Methods

### AO solution

AO (Sigma-Aldrich: No. 318337) solution (100 ppm AO, 0.1 mg/ml) was prepared using the buffer containing 20 mM Tris–HCl, 5 mM EDTA, and 0.1% Triton X-100 at pH 6.8 and filtered using a 0.22-µm filter. Here the pH of the Tris-based buffer was lowered from 8.0 to 6.8 to enhance the red fluorescence [3]. The high EDTA concentration raises the fluorescence of RNA by releasing the latter’s higher-order structures [10]. The AO solution is micro-organism-free due to antibacterial action of EDTA and Tris, and when not exposed to light, is stable for months at room temperature and for years at 4 °C.

### LED lamp and microscope modification

**Fig. 1:**
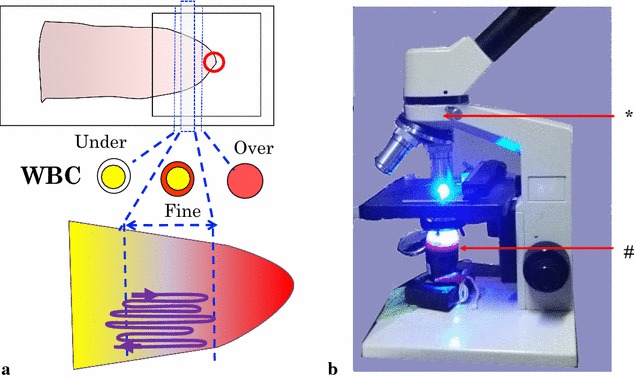
**a** Schema for the revised AO staining and slide examination. The AO droplet (indicated by the red open circle) on a cover slip is placed at the tip of the thin film. A typical scanning route to find parasites is indicated by the meandering line. **b** Picture of a blue LED lamp placed under the stage of a microscope. The LED lamp has a built-in shortpass filter (#) under its condenser lens and a longpass filter (*) is inserted in the microscope body

Starting with a commercially available flashlight comprising condenser lens and a 3-AAA battery compartment, the original lamp was replaced by a 3 W blue LED of 465–475 nm and an interference shortpass filter (VIS 490 nm, Asahi Spectra, Japan) was inserted under the lens to block weak long-wavelength light component with > 520 nm. The battery compartment was removed and the electrodes were connected to an external battery box powered by 3 AA batteries (switch-attached) through electrode clips. This LED lamp was placed under the stage of a microscope. A gelatin longpass filter of 520 nm (SC52, FUJIFILM, Japan) was inserted into the light path of a microscope body to block the excitation light but not AO fluorescence (Fig. 1).

### Sample collection

Blood samples were collected during malariometric surveys on islands in Lake Victoria, Kenya in August 2015. Peripheral blood was collected by finger prick. Thick and thin films were each prepared using 5 µl of blood (total 10 µl) on the same microscope slide. The thin films were made moderately long such that red blood cells were spread as one layer, which is important to obtain good AO staining results. After drying, the thin film was fixed by immersion in 100% ethanol. Blood spots were collected on Whatman 31ET Chr filter paper (Whatman, UK) for DNA extraction and PCR diagnosis.

### AO staining and diagnosis

The AO staining was performed on site using the thin film part of a glass slide as follows (Additional file 1): using a micropipette, a 15-µl droplet of the AO solution was put onto the centre of a 22 × 22 mm cover slip (or 18 µl for a 24 × 24 mm slip), which was placed on a tissue on a bench. The microscope slide was turned upside-down and slowly put onto the cover slip with the tip of the thin film softly adhering to the AO droplet (the position is shown in Fig. 1). The slide and the cover slip were kept upside-down and on a small incline (from 5° to 20° from the horizontal) for 1 min to facilitate the spread of AO solution towards the middle of the thin film, resulting in a decreasing concentration gradient of AO stain.

AO-stained films must be examined without delay before the staining solution under the cover slip dries up. Each thin film was first examined at 100× magnification, starting at the tip of the film, where excess AO caused white blood cells (WBCs) to appear as cells having red nuclei with red cytoplasm. As one moved towards the middle of the thin film (i.e., down the AO concentration gradient), WBCs appeared as yellow nuclei (or green in case of UV excitation) with red cytoplasm. In this narrow band of about 5 mm in width, the search for *Plasmodium* parasites was performed at 400× magnification in a snake-like meandering path (Fig. 1). A malaria parasite is seen as a dichromic image (yellow nucleus and red cytoplasm) in a faint image of red blood cell (RBC). The optimal observation area sometimes spreads further towards the middle of the thin film, where local AO concentration may be too low for WBCs that appear as yellow nuclei without red cytoplasm, but sufficient for malaria parasites. After counting the number of parasitized red blood cells (pRBCs) against 100 WBCs, parasitaemia was determined as the number of pRBCs per WBC and then converted to the number of pRBCs per µl by assuming 8000 WBCs in 1 µl. In high parasitaemia cases, the WBC counts were stopped after detection of 5–10 pRBCs. The cover slips were removed after examination.

### Giemsa diagnosis

The thick films were stained with 3% Giemsa solution for 30 min and examined in a routine manner by experienced microscopists without knowledge of the results from the AO method. After counting the number of parasites against 200 WBCs, parasitaemia was determined as the number of parasites per µl as the same as the AO method.

### PCR diagnosis

DNA was extracted from blood spots on filter paper, and *Plasmodium* infections were detected by PCR as previously described [11].

### Statistical analyses

The sensitivity, specificity, positive predictive value (PPV), and negative predictive value (NPV) of the Giemsa and AO methods were calculated using the PCR results as a standard. For cases that were positive by both the Giemsa and AO methods, the relationship between their respective parasitaemias was evaluated by Pearson’s correlation.

## Results

No staining failure was observed with the revised AO rapid staining and diagnosis on site. On average about 100 samples were examined by one microscopist per day, with examination of each sample taking about 3 min (counting pRBCs against 100 WBCs).

Among 1018 samples (Additional file 2), the numbers of infections detected by the revised AO method using thin films, the Giemsa method using thick films, and PCR were 148, 145, and 306, respectively (Table 1). Among the AO and Giemsa positive cases, 7 and 26 cases were negative by PCR, respectively. Using PCR result as the standard, the AO method demonstrated both a higher sensitivity (46.1 vs 38.9%: p = 0.08) and a higher PPV (95.3 vs 82.1%) than the Giemsa method. The AO method also showed a higher specificity (99.0 vs 96.3%) and a higher NPV (81.0 vs 78.6%) than the Giemsa method, indicating that the former was less likely to detect false positives. The AO diagnosis was more accurate and slightly more sensitive (119%; p = 0.08) than the Giemsa diagnosis.

**Table 1:** Comparison between the AO and Giemsa diagnoses using PCR as the standard

	PCR+ (H, L)	PCR− (H, L)	Total (H, L)
Total	306 (248, 58)	712 (278, 434)	1018 (526, 492)
AO+ (H, L)	141 (120, 21)	7 (7, 0)	148 (127, 21)
AO− (H, L)	165 (128, 37)	705 (271, 434)	870 (399, 471)
Giemsa+ (H, L)	119 (94, 25)	26 (15, 11)	145 (109, 36)
Giemsa− (H, L)	187 (154, 33)	686 (263, 423)	873 (417, 456)

The numbers of cases in high (H) and low (L) transmission settings are shown in parentheses

The performance of the AO and Giemsa methods was compared further by transmission settings. In high transmission settings (N = 526; PCR-positive rate = 47.1%), the AO method had a higher sensitivity (48.4 vs 30.7%; p < 0.02), a higher PPV (94.5 vs 86.2%), a higher specificity (97.5 vs 93.2%), and a higher NPV (67.9 vs 49.2%) than the Giemsa method. In low transmission settings (N = 492; PCR-positive rate = 11.0%), the AO method detected 21 positives (4.3%) without false positives, while the Giemsa method detected 25 positives (5.1%) and 11 false positives. The Giemsa method achieved a higher (though non-significant) sensitivity (43 vs 36%; p > 0.45), but a much lower PPV (69.4 vs 100%), a lower specificity (97.5 vs 100%), and a similar NPV (92.8 vs 92.1%) compared to the AO method. The AO method was more accurate since it achieved higher PPVs and higher specificity than the Giemsa method in both transmission settings.

Among the 92 cases that were positive by both AO and Giemsa staining, a significant correlation (r = 0.77, p < 0.001) was observed between the parasitaemias obtained by the two methods. However the mean parasitaemia by the AO method (12,500/µl) was 61% higher than that by the Giemsa method (7800/µl).

For the 76 PCR-confirmed cases with discordant microscopy results, their distribution based on the pRBC counts by either the AO or the Giemsa method is shown in Table 2. The mean pRBC count of infections missed by the Giemsa method was 107,000/µl, while the mean for those missed by the AO method was 30,600/µl, indicating that the AO method was less likely to misdiagnose these infections. For example, the Giemsa method failed to detect 39 cases which were detected by AO with > 4 pRBCs, while the AO method missed only 7 cases which were detected by Giemsa with > 4 parasites.

**Table 2:** Distribution of PCR-confirmed cases with discordant microscopy diagnosis by pRBC/parasite counts

pRBC/parasite count	1	2	3–4	5–8	9–16	17–32	33–64	65–128	> 128	Total	Mean
AO+/Giemsa−	6	2	2	9	14	7	4	0	5	49	13.4
Giemsa+/AO−	3	10	7	3	2	1	0	0	1	27	3.83

Mean was calculated for all cases except those with > 128 pRBC/parasite

## Discussion

The revised AO diagnosis system was conducted on site in Kenya under difficult conditions (e.g., on a low table under the eaves of a house), nonetheless it detected more *Plasmodium* infections with higher PPVs than the conventional Giemsa method, which was conducted in a clean and well-equipped laboratory. In high transmission settings the AO method was superior to the Giemsa method by all performance metrics. In low transmission settings, the slightly higher sensitivity of the Giemsa method was achieved at the cost of lower PPV due to an excess of false positives. The effect of fatigue from examination of about 100 slides a day might be more pronounced in low transmission settings. Another factor affecting the results would be developer bias, which means that developer subjectivity can affect the microscopic observation. However, this is expected to be minor since about 70% of the microscopic observation was done by a local microscopist who was not involved in the development.

The use of LED as a light source for the AO method is convenient in developing countries with unstable electricity supply. Unlike the halogen lamp used previously [8], the LED lamp used in this study can be powered by batteries and yet is bright enough to use with binocular microscopes after minimal modification. The LED lamp is long-lived and free of maintenance except for the inexpensive (< US$1) AA battery change every 50–100 h. The cost of modification is approximately US$170:-US$150 for the interference shortpass filter and US$20 for the longpass filter, flashlight, blue LED, and battery box. Since LED luminescence usually accompanies with weak long-wavelength components that are too strong to observe AO fluorescence, the expensive shortpass filter is required. The set-up time takes only a few minutes and the modification is non-permanent.

The staining protocol was revised to minimize the occurrence of staining failure. The dichromic range of AO fluorescence is estimated to be 10–20 ppm for cells or 5–15 ppm for bare DNA/RNA from reported data [10]. This narrow range of the optimal concentration together with the high concentration of AO solution [4] often causes staining failure and inconsistency among samples; quantitation of parasitaemia becomes difficult when AO solutions of 1000 ppm [4] and 20 ppm [6] were used with their respective staining protocols. Starting with a standard volume of AO solution on a cover slip, the revised staining protocol produces a directional concentration gradient, such that the properly stained area, where pRBCs are easily identified, can be predictably produced between over- and under-stained areas. This effective concentration-gradient staining technique thus minimizes staining failure. The thick films were not used for this AO diagnosis because they were too thick for the LED light to illuminate the fluorescent dye. In this study, microscopic observation of the thin film stained by AO was finished within 3 min, which is much faster than that of the thick film stained by Giemsa (15–20 min). With the AO method, microscopic observation is performed at 400× magnification, with a visual field that is 2.25 times broader than that at 1000× magnification with the Giemsa diagnosis. Furthermore, malaria parasites are seen more clearly in AO-stained films than in Giemsa-stained films, contributing to the superior performance of the AO method demonstrated in this study.

Since *Plasmodium falciparum* was the predominant species in the study area, the AO method could not be explicitly examined for detection of other *Plasmodium* species. In practice, *Plasmodium vivax* can be easily identified because AO stains the RNA of an infected reticulocyte together with the parasite [3], although the Schüffner’s dots are not stained. As for *Plasmodium malariae* and *Plasmodium ovale*, they are difficult to identify even by Giemsa staining because of their usually low parasitaemia. The inability to use thick films for the AO method also will undermine the identification of very low parasitaemia infections such as may be encountered with these species.

The AO diagnostic system has a problem of recordability: (1) AO fluorescence disappears within a few days after staining; and, (2) re-staining of the thin film is impossible (although secondary AO staining of previously unstained area and Giemsa staining in the AO-stained area without destaining are possible). This point may be compensated by attaching a digital camera to the microscope to capture an image of the field of view. Furthermore, 600× magnification is desired to record morphologies of parasites, although 400× magnification is sufficient to find parasites.

## Conclusion

The revised AO diagnostic system can be readily adopted for use at facilities with existing malaria diagnosis capability by Giemsa staining. The conventional light microscope can be modified for AO diagnosis by inserting a longpass filter in the light path of the microscope body and placing a LED lamp with a built-in shortpass filter under the stage. AO observation is easy to learn. Examination at 400× magnification enables rapid diagnosis, usually within 3 min. Combined with the short staining time, the time-to-diagnosis of the AO method is at least on a par with currently available RDTs (15 min). As a rapid detection method, this AO diagnosis system may be useful for surveillance programmes and treatment follow-ups.

## Additional files

**Additional file 1.** AO Staining procedure.

**Additional file 2.** AO_Giemsa_PCR diagnoses data for 1018 samples.

## Abbreviations

AO: acridine orange
DNA: deoxyribonucleic acid
EDTA: ethylenediaminetetraacetic acid (− 2Na^+^)
HRP2: histidine-rich protein 2
LED: light-emitting diode
PCR: polymerase chain reaction
ppm: parts per million
pRBC: parasitized red blood cell
RBC: red blood cell
RDT: rapid diagnostic test
RNA: ribonucleic acid
Tris: tris hydroxymethyl aminomethane
WBC: white blood cell
PPV: positive predictive value
NPV: negative predictive value
