# Supplementary material for: Improvement of malaria diagnostic system based on acridine orange staining
Source: Malar J. 2018 Feb 7;17:72. doi: 10.1186/s12936-018-2214-8 (PMC5804042; doi:10.1186/s12936-018-2214-8)
Supplement: Supplementary file 1 — Additional file 1. AO Staining procedure. [file 12936_2018_2214_MOESM1_ESM.pptx]

## Slide 1
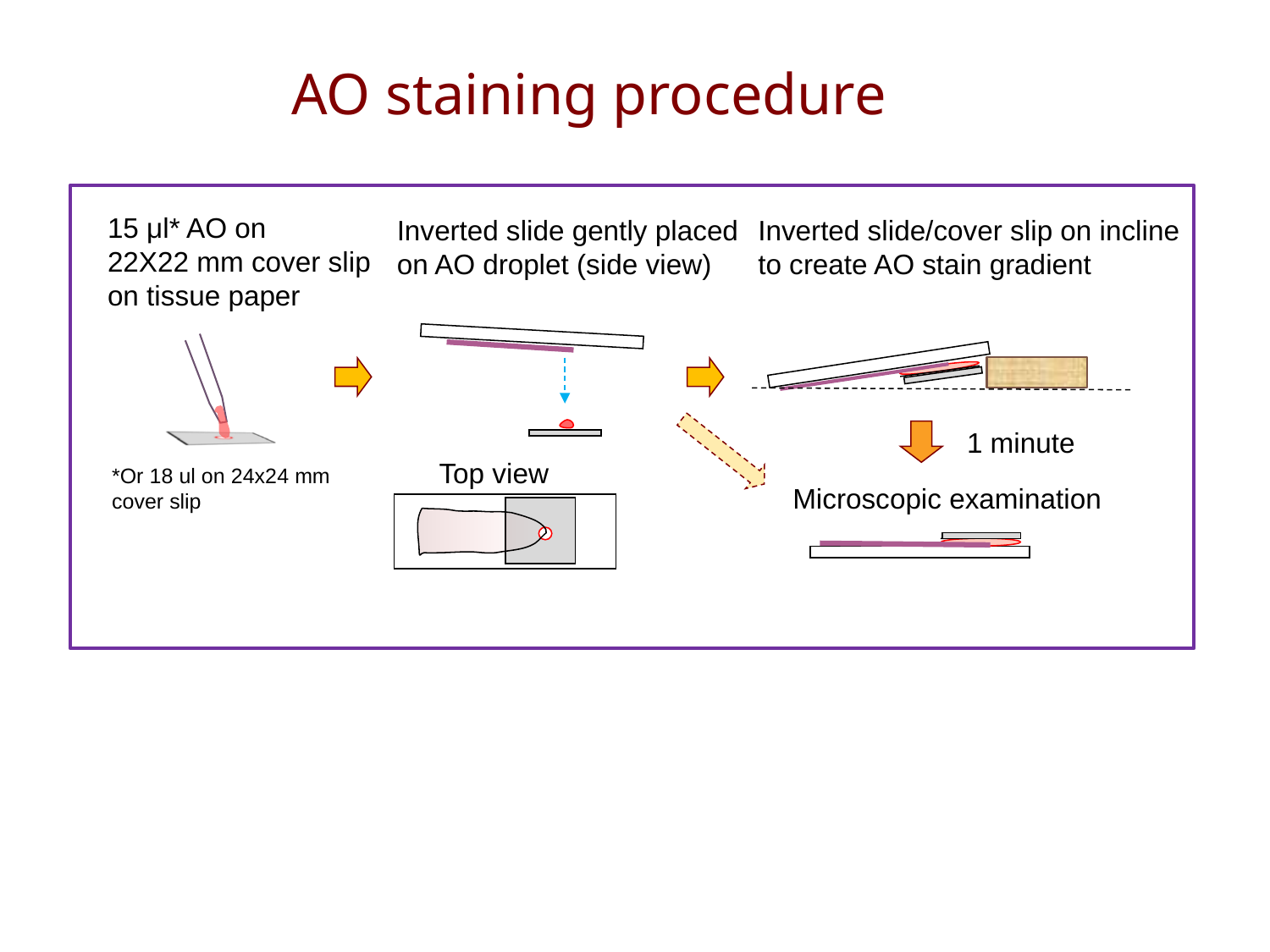

# AO staining procedure
15 μl* AO on
22X22 mm cover slip
on tissue paper
Inverted slide gently placed
on AO droplet (side view)
Inverted slide/cover slip on incline
to create AO stain gradient
Top view
*Or 18 ul on 24x24 mm
cover slip
Microscopic examination
1 minute
